# A direct comparison of selective BH3-mimetics reveals BCL-X_L_, BCL-2 and MCL-1 as promising therapeutic targets in neuroblastoma

**DOI:** 10.1038/s41416-020-0795-9

**Published:** 2020-03-18

**Authors:** Annika Bierbrauer, Maureen Jacob, Meike Vogler, Simone Fulda

**Affiliations:** 1 Institute for Experimental Cancer Research in Pediatrics, Goethe-University Frankfurt, Frankfurt, Germany; 2 German Cancer Consortium (DKTK), Partner Site Frankfurt, Frankfurt, Germany; 3 German Cancer Research Center (DKFZ), Heidelberg, Germany

**Keywords:** Cell biology, Cancer

## Abstract

**Background:**

Despite advances in the treatment of neuroblastoma, patients with high-risk disease still have dismal survival prognosis. Neuroblastoma cells display elevated expression of the antiapoptotic BCL-2 proteins, suggesting that BH3-mimetics may be a promising treatment option. Here, we investigated the role of BCL-2, BCL-X_L_ and MCL-1 in neuroblastoma.

**Methods:**

A panel of neuroblastoma cell lines and primary patient-derived cells were exposed to BH3-mimetics targeting BCL-2 (ABT-199), BCL-X_L_ (A1331852) or MCL-1 (S63845). In addition, protein expression and interaction patterns were analysed using Western blotting and immunoprecipitation.

**Results:**

All tested BH3-mimetics were able to induce apoptosis in neuroblastoma cell lines, indicating that not only BCL-2 but also BCL-X_L_ and MCL-1 may be promising therapeutic targets. Primary patient-derived cells displayed highest sensitivity to A1331852, highlighting the important role of BCL-X_L_ in neuroblastoma. Further analysis into the molecular mechanisms of apoptosis revealed that A1331852 and S63845 displaced proapoptotic proteins like BIM and BAK from their antiapoptotic targets, subsequently leading to the activation of BAX and BAK and caspase-dependent apoptosis.

**Conclusions:**

By using selective BH3-mimetics, this study demonstrates that BCL-2, BCL-X_L_, and MCL-1 are all relevant therapeutic targets in neuroblastoma. A1331852 and S63845 induce rapid apoptosis that is initiated following a displacement of BAK from BCL-X_L_ or MCL-1, respectively.

## Background

Neuroblastoma is the most common tumour in infants of less than a year. It originates from neuroendocrine cells and can develop anywhere in the body along the sympathetic chain.^1^ Clinically, neuroblastoma is highly heterogeneous, with some cases showing spontaneous regression particularly in young patients, but others displaying highly metastatic and progressive disease. The clinical heterogeneity of neuroblastoma makes individual treatment choices difficult, and besides the metastatic state of the tumour also the age of the patients and molecular markers are used in the Children’s Oncology Group (COG) risk stratification. According to the COG classification, patients in the high-risk group have a 5-year survival prognosis of <50%.^2^

Pan-cancer genomic analyses have revealed striking differences between paediatric and adult cancers, with paediatric tumours overall containing a 14 times lower mutational burden.^3^ One common genetic alteration associated with high-risk neuroblastoma and poor prognosis is the amplification of *MYCN* encoding the transcription regulator MYCN.^4^ In contrast to MYC, which is more broadly expressed in adult tissues, MYCN is expressed only in selected tissues and mainly during embryonal development.^5^ By dimerising with MYC-associated factor x, MYCN regulates the transcription of genes involved in multiple cellular processes, including metastasis, angiogenesis and apoptosis.^6^ In addition to *MYCN* amplifications, activating mutations or amplifications of the tyrosine kinase receptor *ALK* have been identified in neuroblastoma.^7–9^ The overall low frequency of genetic alterations in neuroblastoma may be compensated by highly altered epigenetics, which may affect the differentiation status and aggressiveness of neuroblastoma.^10^ In particular, epigenetic silencing of important apoptosis regulators, like caspase-8, has frequently been reported in neuroblastoma.^11^ In addition, inflammatory and survival signals provided by the tumour microenvironment may play an important role in the progression of neuroblastoma and its resistance to apoptosis.^12,13^

Apoptosis can be initiated either by the ligation of death receptors on the plasma membrane or by the release of cytochrome *c* from the mitochondria into the cytosol. This release of cytochrome *c* from the mitochondria is facilitated and regulated by B cell lymphoma 2 (BCL-2) proteins.^14^ Once apoptosis is triggered, the proapoptotic BCL-2 proteins BAX and BAK undergo conformational changes that allow their oligomerisation within the mitochondrial membranes. This activation of BAX and BAK is inhibited by the antiapoptotic BCL-2 proteins, which bind to and sequester BAX and BAK, thus preventing further oligomerisation. BCL-2 homology domain 3 (BH3)-only proteins contribute to apoptosis either by competing with BAX/BAK for the binding of antiapoptotic proteins or by directly interacting with and activating BAX/BAK.

The main antiapoptotic BCL-2 proteins, BCL-2, BCL-X_L_ and MCL-1, are frequently overexpressed in many cancer types and ensure cancer cell survival during cellular stress.^15,16^ Soon after its initial discovery, high expression of BCL-2 has been identified in some neuroblastoma tissues, which was confirmed in multiple studies.^17–19^ Also, BCL-X_L_ and MCL-1 are highly expressed in neuroblastoma and may prevent apoptosis induction upon chemotherapy treatment.^20,21^ Therefore, all three main antiapoptotic BCL-2 proteins may represent potential targets for the development of novel therapeutic options in neuroblastoma. To target and inhibit the antiapoptotic BCL-2 proteins, several small-molecule inhibitors called BH3-mimetics have been developed that either target multiple antiapoptotic BCL-2 proteins or display specificity for only one target. Thereby, selective BH3-mimetics may have the advantage of displaying less toxicity on healthy cells.^22^ With the clinical approval of ABT-199/Venetoclax, a highly potent selective inhibitor of BCL-2 for the treatment of leukaemia, these BH3-mimetics are emerging as powerful new assets in the fight against cancer.^22,23^ Besides ABT-199, highly potent inhibitors have been discovered, which in the case of A1331852 selectively inhibit BCL-X_L_ or in the case of S63845 selectively target MCL-1,^24,25^ thus allowing efficient inhibition of all main antiapoptotic BCL-2 proteins. However, to prevent unwanted toxicities, it is essential to identify the most effective BH3-mimetic in a given tumour type and tailor the use of BH3-mimetics to patients most likely to achieve benefits. This is particularly important when targeting BCL-X_L_, as BCL-X_L_ is an essential antiapoptotic protein in platelets and inhibition of BCL-X_L_ caused thrombocytopenia.^26^

In this study, we compared the effects of selective BH3-mimetics in a panel of neuroblastoma cell lines and primary-derived cells, with the aim to assess which antiapoptotic BCL-2 protein is the most important therapeutic target in neuroblastoma.

## Methods

### Neuroblastoma cells

CHLA-15, CHLA-20, SMS-KCNR and Lan-6 cells were provided by the COG of the National Cancer Institute. NLF cells were kindly provided by Jindrich Cinatl.^27^ SJNB-12 cells were kindly provided by Catrin Pritchard (University of Leicester). All other cell lines were provided by ATCC (SK-N-SH, SK-N-AS, SH-EP, CHP-212) or DSMZ (Lan-5, Kelly, IMR-32, SK-N-BE (2)). Cell lines were cultured in DMEM (Dulbecco’s modified Eagle’s medium) or RPMI-1640 medium (Life Technologies Inc., Darmstadt, Germany) supplemented with 1 mmol/L glutamine, 1% penicillin/streptomycin, 25 mmol/L HEPES and 10% or 20% foetal calf serum (FCS) (all from Invitrogen, Karlsruhe, Germany). For culturing CHLA-15 cells, 1% insulin-transferin-sellenium was added. Lan-5 and NLF cells were cultured in IMDM (Iscove’s modified Dulbecco’s medium) supplemented with 10% FCS and 1% penicillin/streptomycin. All cell lines were authenticated by short tandem repeat profiling and routinely tested to ensure mycoplasma-free cultures. Primary neuroblastoma cells were obtained at the Ludwig-Maximilians University of Munich with patient consent and local ethic committee approval. Cells were isolated by mechanical disaggregation from surgical specimens obtained from patients with neuroblastoma and cultured in DMEM supplemented with 1 mmol/L glutamine, 1% penicillin/streptomycin, 25 mmol/L HEPES, and 20% FCS as described previously.^28^

### Measurement of cell death

Cells were treated with indicated concentrations of ABT-199 (Selleck Chemicals, Houston, TX), A1331852 (Selleck) or S63845 (Appexbio, Taiwan) prior to analysis of death by staining with propidium iodide and microscopic analysis using ImageXpress (Molecular Devices, Biberach, Germany). A representative image of this analysis is displayed in Supplementary Fig. 1. Alternatively, cell death was investigated by flow cytometric analysis of FSC/SSC (forward/side scatter) properties using FACScanto II (BD Bioscience, Heidelberg, Germany). Viability was assessed using CellTiterGlo (Promega, Mannheim, Germany) and Tecan Infinite M200 plate reader. Loss of mitochondrial membrane potential (MMP) was investigated following staining with 100 nM TMRM (tetramethylrhodamine methyl ester) (Thermo Fisher, Waltham, MA) and flow cytometry. To inhibit caspases, the broad-range caspase inhibitor *N*-benzyloxycarbonyl-Val-Ala-Asp-fluoromethylketone (zVAD.fmk) (Bachem, Heidelberg, Germany) was used.

### Western blotting and immunoprecipitation

For analysis of protein expression, cells were lysed in 0.5% Triton X buffer. Western blotting was performed using the following antibodies: mouse anti-BCL-2 (Dako, M088701-2, Hamburg, Germany), rabbit anti-BCL-X_L_ (Cell Signaling, 2762S, Beverly, MA), rabbit anti-MCL-1 (Enzo, ADI-AAP-240F, Farmindale, NY), rabbit anti-BCL-w (Cell Signaling, 2724S), rabbit anti-BIM (Cell Signaling, 3183S), mouse anti-NOXA (Enzo, ALX-804-408), rat anti-BMF (Enzo, ALX-804-343), rabbit anti-BAK (Upstate/Merck, 06-536), mouse anti-BAX (BD Bioscience, 610983), rabbit anti-PUMA (Cell Signaling, 4976S), rabbit anti-caspase-3 (Cell Signaling, 9662S), mouse anti-PARP (poly(ADD)ribose polymerase) (Cell Signaling, 9546S), mouse anti-β-actin (Sigma, A5441, Deisenhofen, Germany) or mouse anti-GAPDH (glyceraldehyde 3-phosphate dehydrogenase) (BioTrend, 5G4-6C5). For immunoprecipitation, proteins were extracted in 1% CHAPS (3-((3-cholamidopropyl) dimethylammonio)-1-propanesulfonate) buffer. Activation of BAX and BAK was assessed by immunoprecipitation with conformation-specific mouse anti-BAX clone 6A7 antibody (Sigma) or mouse anti-BAK AB-1 antibody (Calbiochem). The following antibodies were used for the detection of interaction between pro- and antiapoptotic proteins: rabbit anti-MCL-1 (Enzo, ADI-AAP-240F), hamster anti-BCL-2 (BD Bioscience, 551051), rabbit anti-BCL-X_L_ (Abcam, ab32370) and rabbit anti-BAK (Abcam, ab32371). Antibodies were crosslinked with dimethyl pimelimidate (Sigma) to Dyna protein G beads (Thermo Fisher) before precipitation overnight.

### Silencing of BIM

For knockdown of the BH3-only protein BIM, cells were electroporated using Neon transfection system (Thermo Fisher) with two pulses of 20 ms at 1200 V and 100 nM of silencer select small interfering RNAs (siRNAs) (#1s195011, #2s195012) (Thermo Fisher). Non-targeting siRNA was used as a control (#4390843).

### Statistics

Quantification of protein expression was performed using the ImageJ 3.1 software. EC_50_ values and linear regression were calculated using GraphPad Prism. Statistical significance was calculated in Excel using *t* test.

## Results

### BCL-2, BCL-X_L_ and MCL-1 are promising therapeutic targets in neuroblastoma

**Fig. 1:**
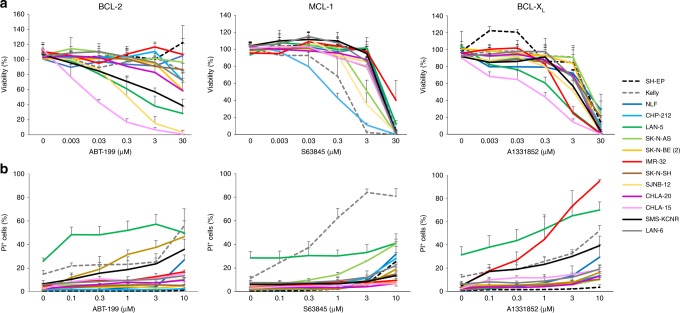
Neuroblastoma cells display heterogeneous sensitivity to BH3-mimetics. Neuroblastoma cells were treated with indicated concentrations of ABT-199, S63845 or A1331852. **a** Viability was assessed at 72 h using CellTiterGlo assay and normalised to untreated control cells. **b** Cell death as indicated by the percentage of PI-positive cells was assessed at 48 h by microscopy. Data shown are mean ± SD (*n* = 3–5).

To evaluate the role of the different antiapoptotic BCL-2 proteins and investigate the potential of BH3-mimetics in neuroblastoma, we directly compared the efficacy of ABT-199, S63845 and A1331852 in neuroblastoma cell lines. Our panel comprised 14 cell lines with diverse genetic and morphological characteristics and included several cell lines with adverse prognostic markers like *MYCN* amplification, *ALK* mutation or *TP53* mutation (Supplementary Table 1). Notably, all BH3-mimetics were able to induce loss of viability (Fig. 1a) and cell death (Fig. 1b) in selected cell lines. A direct comparison of the efficacy of the different compounds as calculated from viability data revealed that three out of 14 cell lines were sensitive to ABT-199 at concentrations below 3 μM (SJNB-12, CHLA-15 and Lan-5) and, similarly, three out of 14 responded to S63845 (SJNB-12, Kelly and CHP-212) and three out of 14 displayed sensitivity to A1331852 (IMR-32, Lan-5 and CHLA-15), highlighting that besides BCL-2, also MCL-1 and BCL-X_L_ may be promising therapeutic targets in neuroblastoma. These data also revealed that some neuroblastoma cells were sensitive to multiple BH3-mimetics (SJNB-12, Lan-5 and CHLA-15) and hence depend on different antiapoptotic BCL-2 proteins for survival, whereas others only responded to one selective BH3-mimetic (IMR-32, CHP-212 and Kelly). Taken together, these data highlight that BCL-X_L_, BCL-2 and MCL-1 all possess essential antiapoptotic functions in neuroblastoma.

### Expression of BCL-2 but not BCL-X_L_ or MCL-1 correlates with sensitivity to BH3-mimetics

**Fig. 2:**
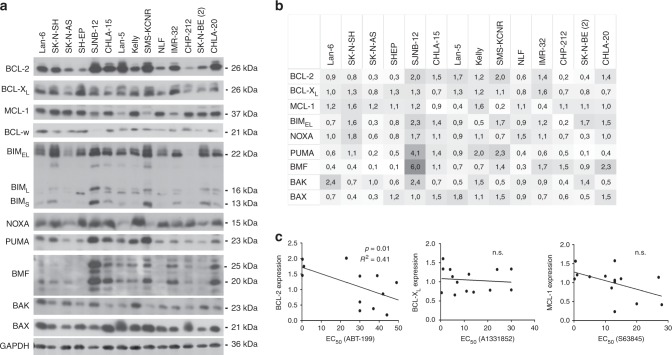
Neuroblastoma cells have high expression of all main BCL-2 proteins. **a** Expression of BCL-2 proteins in neuroblastoma cell lines was assessed by Western blotting. One representative experiment is shown (*n* = 3). GAPDH was used as a loading control. **b** For quantification, three independent experiments were analysed by densitometry to calculate mean expression of BCL-2 family proteins versus loading control. Data are shown as a heatmap. **c** Linear regression analysis demonstrates a correlation between BCL-2 expression and sensitivity to ABT-199, as assessed by calculation of EC_50_ values based on the CellTiterGlo data presented in Fig. 1a. Linear regression analysis indicates no significant correlation between BCL-X_L_ levels and sensitivity to A1331852 or MCL-1 levels and sensitivity to S63845. n.s. Not significant.

Next, we investigated the expression of BCL-2 proteins in neuroblastoma cell lines (Fig. 2a, b). Western blot analysis revealed that BCL-2 expression was very heterogeneous in neuroblastoma cell lines, with some cell lines displaying relatively high BCL-2 protein levels (SJNB-12, Lan-5 and SMS-KCNR), and others having relatively low expression (SH-EP, CHP-212). In addition, most cell lines expressed relatively high levels of MCL-1, BCL-X_L_ and BCL-w. Neuroblastoma cells also displayed relatively high expression of BH3-only proteins like BIM, PUMA and NOXA, with strikingly high expression of BMF in the SJNB-12 cells. The pore-forming multidomain proteins BAX and BAK were expressed in all cell lines, demonstrating that overall the apoptotic machinery is intact in neuroblastoma cells. Of note, in this analysis the expression of all BCL-2 proteins was independent of *MYCN* status (Fig. 2b and Supplementary Table 1).

To assess whether high expression of the targeted antiapoptotic BCL-2 proteins may correlate with sensitivity to BH3-mimetics, the expression of antiapoptotic BCL-2 proteins was quantified and linear regression analysis was performed. High expression of BCL-2 significantly correlated with sensitivity to ABT-199 (Fig. 2c), highlighting the potential of BCL-2 expression to serve as a biomarker to predict responses to ABT-199.

In contrast, the expression of BCL-X_L_ or MCL-1 was not associated with the response to A1331852 or S63845, respectively (Fig. 2c), suggesting that besides expression of the targets also other factors determine sensitivity to inhibitors of BCL-X_L_ or MCL-1.

### Neuroblastoma cells are highly primed to undergo apoptosis

Besides the expression of the targeted proteins, the response to BH3-mimetics may correlate with the amount of proapoptotic proteins like BIM sequestered in complexes with the antiapoptotic BCL-2 proteins. Thereby, cells with high levels of sequestered proapoptotic proteins are primed to undergo apoptosis when this interaction is disrupted by BH3-mimetics, thus leading to a release of proapoptotic BCL-2 proteins and initiation of mitochondrial outer membrane permeabilisation (MOMP). To investigate whether neuroblastoma cells display complex formation between pro- and antiapoptotic BCL-2 proteins, immunoprecipitations of the antiapoptotic proteins BCL-2, BCL-X_L_ and MCL-1 were performed in seven selected cell lines with a range of sensitivities (Supplementary Fig. 2). The cells showing the highest sensitivity to ABT-199 (SJNB-12 and SMS-KCNR) expressed high levels of BIM bound to BCL-2. However, also the ABT-199-resistant cells IMR-32 and CHLA-20 displayed high binding of BIM to BCL-2, indicating that binding of BIM does not necessitate susceptibility to ABT-199. Some BIM_EL_ was also bound to BCL-X_L_, in particular in the IMR-32 cells, which had high sensitivity to A1331852. BIM binding to MCL-1 was most pronounced in NLF, Kelly and SK-N-AS cells. Taken together, these experiments showed that all antiapoptotic BCL-2 proteins can bind to and sequester BIM, with overall highest binding of BIM detected in complex with BCL-2. The extent of BIM binding to the antiapoptotic BCL-2 proteins was only partially associated with a response to selective BH3-mimetics, since BIM was also bound to some antiapoptotic BCL-2 proteins in cells that did not respond to BH3-mimetics. In line with its published binding profile,^8^ the BH3-only protein NOXA was exclusively bound by MCL-1, but not by BCL-2 or BCL-X_L_.

### A1331852 and S63845 induce intrinsic apoptosis in selected neuroblastoma cell lines

**Fig. 3:**
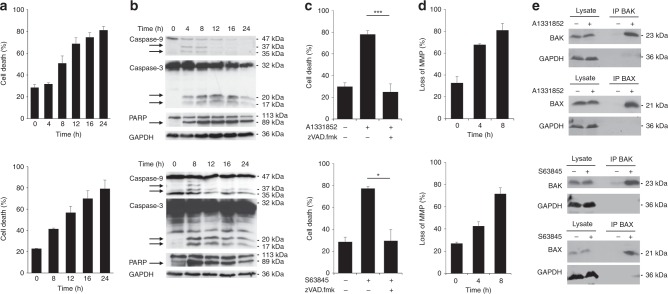
Inhibition of BCL-X_L_ or MCL-1 leads to intrinsic apoptosis. IMR-32 cells were treated with 1 μM A1331852 (upper panels), and Kelly cells were treated with 1 μM S63845 (lower panels). **a** Kinetics of cell death were investigated by flow cytometry and gating of FSC/SSC. Data shown are mean ± SD (*n* = 3). **b** Cleavage of caspases and PARP was analysed by Western blotting. **c** Addition of the caspase inhibitor zVAD.fmk (25 μM) prevented cell death as assessed at 24 h by flow cytometric analysis and gating of FSC/SSC. Data shown are mean ± SD (*n* = 3). *P < 0.05; ***p < 0.001. **d** Loss of MMP was investigated by staining with TMRM and flow cytometry. Data shown are mean ± SD (*n* = 3). **e** Cells were treated for 4 h before lysis in CHAPS buffer. Activation of BAK and BAX was investigated using immunoprecipitation (IP) with specific antibodies that recognise the conformational change of BAX or BAX during activation. One representative experiment is shown (*n* = 2).

The molecular mechanisms of cell death induced by ABT-199 in neuroblastoma have already been elucidated in previous studies;^29,30^ however, so far the mechanisms of cell death induced by selective inhibitors of BCL-X_L_ or MCL-1 have not been investigated. To study how inhibition of BCL-X_L_ or MCL-1 may initiate cell death in neuroblastoma, we selected cell lines sensitive to A1331852 (IMR-32, Fig. 3, upper panels) or S63845 (Kelly, Fig. 3, lower panels). Treatment with A1331852 or S63845 resulted in time-dependent induction of cell death already after 8 h of treatment (Fig. 3a). Cleavage of caspase-9 and -3 was induced by A1331852 or S63845 after 4 to 8 h of treatment, and led to pronounced cleavage of the caspase substrate PARP, demonstrating activation of the intrinsic apoptotic pathway (Fig. 3b). Treatment with A1331852 induced dose-dependent exposure of phosphatidylserine, a hallmark of apoptosis (Supplementary Fig. 3). To examine whether cell death induced by A1331852 or S63845 was caspase dependent, we utilised the caspase inhibitor zVAD.fmk, which caused a significant inhibition of cell death (Fig. 3c). Since the intrinsic apoptotic pathway is initiated at the mitochondria following MOMP and loss of MMP, we asked whether A1331852 and S63845 induced a loss of MMP (Fig. 3d). A1331852 induced a pronounced loss of MMP already after 4 h of treatment, highlighting how rapid cell death is initiated following BCL-X_L_ inhibition. S63845 induced a loss of MMP at slightly slower kinetics, with a minor loss of MMP observed after 4 h, which was further increased after 8 h of treatment. MOMP is mediated by conformational changes of the pore-forming BCL-2 proteins BAX and BAK, which insert into the mitochondrial membrane and oligomerise within the membrane, leading to pore formation and loss of MMP. To assess whether A1331852 and S63845 induced BAX and BAK activation, immunoprecipitation was performed using antibodies that specifically detect the conformational change during the activation of BAX and BAK (Fig. 3e). A1331852 and S63845 induced activation of both BAK and BAX already after 4 h of treatment. Taken together, these data show that A1331852 and S63845 induced activation of BAX and BAK, followed by the initiation of the intrinsic apoptotic caspase cascade and caspase-dependent apoptosis.

### BH3-mimetics cause a displacement of proapoptotic proteins from antiapoptotic BCL-2 proteins

**Fig. 4:**
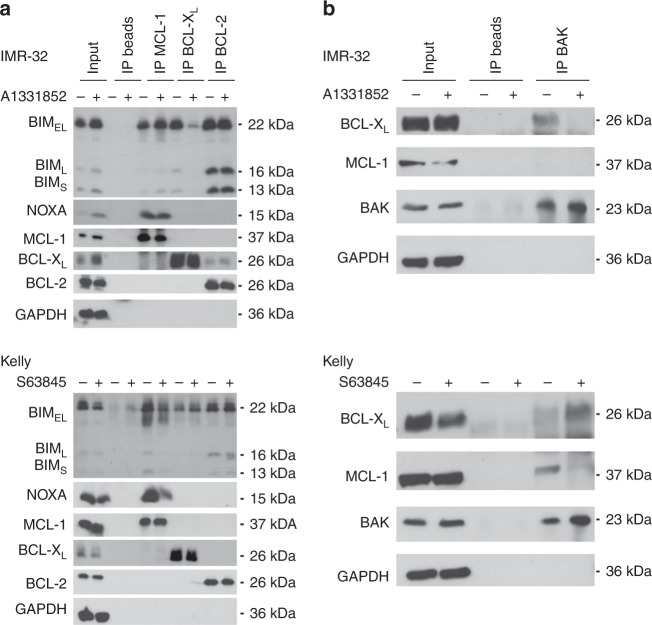
BH3-mimetics displace BH3-only proteins from antiapoptotic BCL-2 proteins. **a** IMR-32 cells were treated with A1331852 (1 μM) and Kelly cells were treated with S63845 (1 μM) for 4 h before lysis in CHAPS buffer. Interaction of pro- and antiapoptotic BCL-2 proteins was investigated by IP of antiapoptotic BCL-2 proteins MCL-1, BCL-X_L_ or BCL-2 upon treatment with BH3-mimetics. **b** IMR-32 cells were treated with 1 μM A1331852 (upper panel) and Kelly cells were treated with 1 μM S63845 (lower panel) for 4 h before lysis in CHAPS buffer. Interaction of BAK with antiapoptotic BCL-2 proteins was investigated by IP with anti-BAK antibody. GAPDH is shown as a loading control.

Activation of BAX and BAK is regulated via a direct inhibition and sequestration by the antiapoptotic BCL-2 proteins, but also by an interaction with BH3-only proteins like BIM and PUMA. In the latter mode, when released from antiapoptotic BCL-2 proteins, the activator BH3-only proteins may directly bind BAX and BAK, thereby inducing the conformational change required for the activation and oligomerisation of BAX and BAK. Since we observed high binding of BIM to the antiapoptotic BCL-2 proteins, we asked whether treatment with BH3-mimetics may displace and release BIM from the antiapoptotic BCL-2 proteins. In IMR-32 cells, BIM_EL_ was bound to all main antiapoptotic BCL-2 proteins, whereas BIM_L_ and BIM_S_ were predominantly bound by BCL-2 and only a minor amount was bound by BCL-X_L_ (Fig. 4a). A1331852 induced a displacement of all three BIM isoforms from BCL-X_L_, whereas the amount of BIM sequestered by BCL-2 or MCL-1 was not affected, confirming on-target activity of A1331852. In Kelly cells, BIM is predominantly sequestered by MCL-1 and BCL-2 with moderate binding to BCL-X_L_. Treatment with S63845 induced displacement of all three BIM isoforms as well as NOXA from MCL-1, in line with on-target activity of S63845. Taken together, these studies demonstrate that the selective BH3-mimetics A1331852 and S63845 displace BH3-only proteins from their antiapoptotic targets, and that these BH3-only proteins may now be available to activate BAX and BAK.

To investigate whether the displacement of BIM from antiapoptotic BCL-2 proteins is required for cell death induced by BH3-mimetics, siRNA-mediated silencing of BIM was performed. However, individual silencing of BIM had a minor impact on cell death induced by BH3-mimetics, indicating that BIM is not the sole mediator of cell death (Supplementary Fig. 4).

Since BH3-mimetics may activate BAK by displacing BAK from antiapoptotic BCL-2 proteins, we next investigated the influence of BH3-mimetics on a direct sequestration of BAK by BCL-X_L_ and/or MCL-1. To this end, we performed immunoprecipitation of BAK (Fig. 4b). In IMR-32 cells, BAK was already complexed with BCL-X_L_, but not with MCL-1. Treatment with A1331852 abrogated the interaction of BAK with BCL-X_L_, indicating that in IMR-32 cells an essential pro-survival function of BCL-X_L_ was to sequester BAK and that inhibition of BCL-X_L_ displaced BAK, thus allowing its oligomerisation. By comparison, in Kelly cells, BAK was primarily complexed with MCL-1. Inhibition of MCL-1 by S63845 released BAK from MCL-1, resulting in redistribution of some of the released BAK to BCL-X_L_. In conclusion, these studies indicate that the antiapoptotic BCL-2 proteins maintain survival of neuroblastoma cells by inhibiting BAK and that treatment with BH3-mimetics released BAK from its antiapoptotic binding partners.

### Evaluation of BH3-mimetics in primary patient-derived neuroblastoma cells

**Fig. 5:**
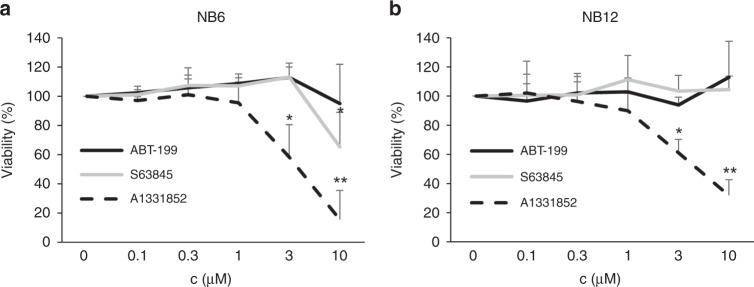
Inhibition of BCL-X_L_ reduces viability of primary neuroblastoma cells. Primary-derived neuroblastoma cells (NB6 and NB12), which are not *MYCN* amplified, were exposed to different concentrations of ABT-199, A1331852 and S63845. **a** Viability of fast-growing NB6 cells was analysed by CTG assay after 3 days of culture. **b** Viability of slow-growing NB12 cells was analysed by CTG assay after 7 days of culture. Data shown are mean ± SD (*n* = 4). *P < 0.05; **p < 0.01.

To evaluate our findings in model systems that may be more relevant for clinical applications than established cell lines, we extended our studies to primary cells derived from neuroblastoma patients. Treatment of primary cells with the selective BH3-mimetics ABT-199, A1331852 and S63845 revealed a prominent role for BCL-X_L_ in maintaining neuroblastoma cell survival (Fig. 5). Surprisingly, in both samples available for testing, A1331852 induced a loss of viability at 3 and 10 μM, whereas ABT-199 did not show any effect. The MCL-1 inhibitor S63845 induced some loss of viability in NB6 cells at 10 μM, confirming that MCL-1 may also be a relevant therapeutic target in neuroblastoma.

## Discussion

Since the survival prognosis of high-risk neuroblastoma patients remains poor, better treatment options are urgently required, and several novel agents are currently being investigated in clinical trials.^31^ However, patient numbers are relatively low and clinical trials, in particular those for targeted agents, need therefore to be stratified and aimed at the patient population most likely to benefit from treatment.^32^ Clinical responses observed in haematological malignancies indicate that BH3-mimetics like ABT-199 may act independently of adverse genetic markers, highlighting their potential to treat high-risk patient groups with limited treatment options.^33^ Notably, ABT-199 is currently also being investigated in clinical trials for the treatment of relapsed or refractory neuroblastoma (NCT03236857).^34^ In line with our data, previous preclinical studies have demonstrated that selected neuroblastoma cell lines express high BCL-2 protein levels and display high sensitivity towards ABT-199.^29,30,35^ However, BH3 profiling, an assay investigating dependencies on antiapoptotic BCL-2 proteins and hence serving as a surrogate biomarker for sensitivity towards BH3-mimetics,^36^ has also highlighted that neuroblastoma is heterogeneous in regards to the BCL-2 family, and that not all cases of neuroblastoma rely on BCL-2 for survival.^37^ Here, we show that susceptibility to ABT-199 correlates with BCL-2 protein expression. Sensitivity to BCL-2 inhibitors has previously been associated with amplification of *MYCN* and high expression of NOXA.^30^ In our panel of cell lines, we do not observe an association between *MYCN* status and sensitivity to ABT-199 or expression levels of NOXA, but the number of cell lines included in our study may be too low for statistical analysis.

Besides BCL-2, also MCL-1 is emerging as an additional target in multiple cancer entities including neuroblastoma,^15,29^ and high MCL-1 protein expression was observed in the majority of neuroblastomas.^21^ Further support for an important role of MCL-1 in neuroblastoma was provided by knockdown experiments showing that siRNA-mediated loss of MCL-1 was sufficient to induce apoptosis in three neuroblastoma cell lines.^21^ The role of BCL-X_L_ in neuroblastoma has been less studied, but early studies have found high expression in the majority of cell lines and have linked the expression of BCL-X_L_ with chemoresistance.^20^ The recent development of selective and potent inhibitors of MCL-1^24^ and BCL-X_L_^25^ has enabled a direct comparison of the main antiapoptotic BCL-2 proteins and their role in apoptosis resistance. Here, we provide the first side-by-side comparison of ABT-199, S63845 and A1331852 in neuroblastoma cell lines and primary-derived cultures and find that all three antiapoptotic BCL-2 proteins are relevant therapeutic targets. Rather surprisingly, with three out of 14 cell lines responding to A1331852, three out of 14 being sensitive to ABT-199 and three out of 14 responding to S63845, our data indicate that BCL-X_L_, BCL-2 and MCL-1 are equally prevalent therapeutic targets. Previous studies have mainly been performed with the BCL-2/BCL-X_L_ inhibitors ABT-737 and ABT-263, and the sensitivity of neuroblastoma cells to these BH3-mimetics has largely been attributed to an inhibition of BCL-2 rather than BCL-X_L_.^38–40^ Of note, SMS-KCNR cells, which have previously been described to be sensitive to ABT-737 due to high BCL-2 expression,^40^ are more sensitive to A1331852 than to ABT-199 in our study, indicating that both BCL-2 and BCL-X_L_ may be important for cellular survival. The high susceptibility of neuroblastoma cells towards A1331852 observed in our study indicates that BCL-X_L_ may play a more important role in neuroblastoma than previously anticipated. To our knowledge, this is the first time that a potent and selective inhibitor of BCL-X_L_ has been investigated in neuroblastoma. Of note, we also observed higher sensitivity towards A1331852 than towards ABT-199 in both primary patient-derived cell cultures, highlighting the translational relevance of our study. Inhibition of BCL-X_L_ has been identified as a potent senolytic strategy, and thus treatment with A1331852 may have the additional benefit of targeting senescent cells.^41,42^ Taken together, these findings underscore the importance of BCL-X_L_ as therapeutic target in neuroblastoma. Hence, clinical trials with BH3-mimetics may be more promising when a dual BCL-2/BCL-X_L_ inhibitor like ABT-263 is used rather than ABT-199. Of note, the concentrations of BH3-mimetics required to induce apoptosis in neuroblastoma cells are in the low micromolar range, with some cell lines displaying sensitivity to <1 μM. For ABT-199, with current dosing schedules, plasma concentrations in adult patients are in the range of 1–3 μM,^33^ indicating that sufficient drug exposure may be achievable. However, clinical trials in paediatric patients are currently ongoing,^34^ and pharmacokinetic and toxicity data for children are still unknown. A well-described on-target toxicity of BCL-X_L_ inhibitors is an effect on mature platelets, since these rely on BCL-X_L_ for survival and inhibition of BCL-X_L_ causes platelet apoptosis.^26^ Hence, patients treated with ABT-263 experienced thrombocytopenia as dose-limiting toxicity.^43^ However, BCL-X_L_ is particularly important in mature platelets, whereas megakaryocytes and the generation of new platelets were not affected, resulting in an overall younger and more reticulated platelet population.^44^ Notably, none of the patients experienced severe bleeding events, and it remains unclear how well an inhibitor of BCL-X_L_ would be tolerated by paediatric patients.

In contrast to the response to ABT-199, which correlated with high expression of BCL-2, we found no association between expression levels of BCL-X_L_ and sensitivity to A1331852. However, we found high binding of proapoptotic BCL-2 proteins to BCL-X_L_ in cells that were sensitive to A1331852, indicating that the interaction between different BCL-2 proteins rather than their expression levels determine sensitivity to A1331852. In this regard, both BIM and BAK were highly bound by BCL-X_L_ and displaced by A1331852, but silencing of BIM had little influence on apoptosis induced by A1331852. BIM has previously been indicated as an essential mediator of apoptosis in neuroblastoma,^45^ but our data indicate that BH3-mimetics may induce apoptosis also independently of BIM. Thus, we hypothesise that in neuroblastoma cells the main function of BCL-X_L_ may be to sequester already partially activated BAK.

With three out of 14 cell lines responding to S63845, the inhibition of MCL-1 as a stand-alone approach also appears promising in neuroblastoma. Additionally, several studies have implicated MCL-1 as an important mediator of cell survival, in particular in the context of resistance to other anti-cancer drugs including BH3-mimetics.^29,39,46^ Therefore, additional studies are required to investigate the potential of combining selective BH3-mimetics and assess the potential of MCL-1 as resistance factor. Interestingly, the sensitive cell line Kelly displayed high binding of BAK to MCL-1, which was disrupted by S63845. As BIM appeared to be not required for S63845-induced apoptosis, this displacement of BAK from MCL-1 may be sufficient to initiate apoptosis without the need for BH3-only proteins, as recently suggested by others.^47^ Therefore, inhibition of MCL-1 may be therapeutically relevant in particular in combination treatments, where cellular stress may result in partial activation of BAK. In this situation, MCL-1 may play an essential role in sequestering partially active BAK, thus representing a novel Achilles heel for cancer cell survival and opening up novel applications of MCL-1 inhibitors like S63845 for the treatment of neuroblastoma. Therefore, one important aspect that requires further investigation is to understand what drives this partial BAK activation and determines whether partially active BAK is sequestered by BCL-X_L_ or MCL-1.

To translate BH3-mimetics into clinical treatments further studies, especially in freshly isolated primary patient-derived cells, are needed to explore sensitivity to BH3-mimetics and identify predictive biomarkers that may help to recruit patients into clinical trials. Therefore, the development of assays assessing BH3-mimetic parsing with small biopsy samples would be beneficial. Taken together, this study provides the first detailed characterisation of selective BH3-mimetics in neuroblastoma and identifies all three main antiapoptotic BCL-2 proteins as important therapeutic targets. Molecular analysis of the induction of apoptosis indicates that sensitivity to ABT-199 is associated with high expression of BCL-2, whereas sensitivity to A1331852 or S63845 was independent of protein expression. In sensitive cell lines, a sequestration of BAK by BCL-X_L_ or MCL-1, respectively, was disrupted by BH3-mimetics, thus leading to mainly BIM-independent apoptosis. These findings have important implications for the development of therapeutic approaches to target antiapoptotic BCL-2 proteins in neuroblastoma.

## Supplementary information

Supplemental Material

## Data Availability

Mutation data displayed in Supplementary Table 1 were derived from the publicly available data sources COSMIC and CCLE. All data generated or analysed during this study are included in this article and its supplementary information files.
