# Supplementary material for: A direct comparison of selective BH3-mimetics reveals BCL-XL, BCL-2 and MCL-1 as promising therapeutic targets in neuroblastoma
Source: Br J Cancer. 2020 Mar 18;122(10):1544–51. doi: 10.1038/s41416-020-0795-9 (PMC7217842; doi:10.1038/s41416-020-0795-9)
Supplement: Supplementary file 1 — Supplemental Material [file 41416_2020_795_MOESM1_ESM.pdf]

Supplementary Table 1

	Genetics			EC_{50} (μM)		
**Name**	**NMYC**	**ALK**	**TP53**	**ABT-199**	**S63845**	**A1331852**
Lan-6	wt	mut	wt	>30	10	7
SK-N-SH	wt	mut	wt	>30	13	24
SK-N-AS	wt	wt	wt	>30	5	21
SH-EP	wt	n.d.	wt	>30	27	>30
SJNB-12	wt	mut	wt	0.25	1	4
CHLA-15	wt	mut	wt	0.02	12	0.4
CHLA-20	wt	mut	wt	>30	13	6
Lan-5	amp	mut	wt	1	20	1
Kelly	amp	mut	mut	>30	0.5	12
SMS-KCNR	amp	mut	wt	23	13	5
NLF	amp	wt	wt	>30	15	>30
IMR-32	amp	wt	wt	>30	28	1
CHP-212	amp	wt	wt	>30	0.18	14
SK-N-BE(2)	amp	wt	mut	>30	13	11

Supplementary Table 1: Characteristics of neuroblastoma cell lines used in this study.
Status for *MYCN* amplification or *ALK* mutation as well as the EC_{50} values for BH3-mimetics are presented for all neuroblastoma cell lines used in this study. EC_{50} values were calculated from viability data presented in Figure 1A.

Supplementary Figure 1

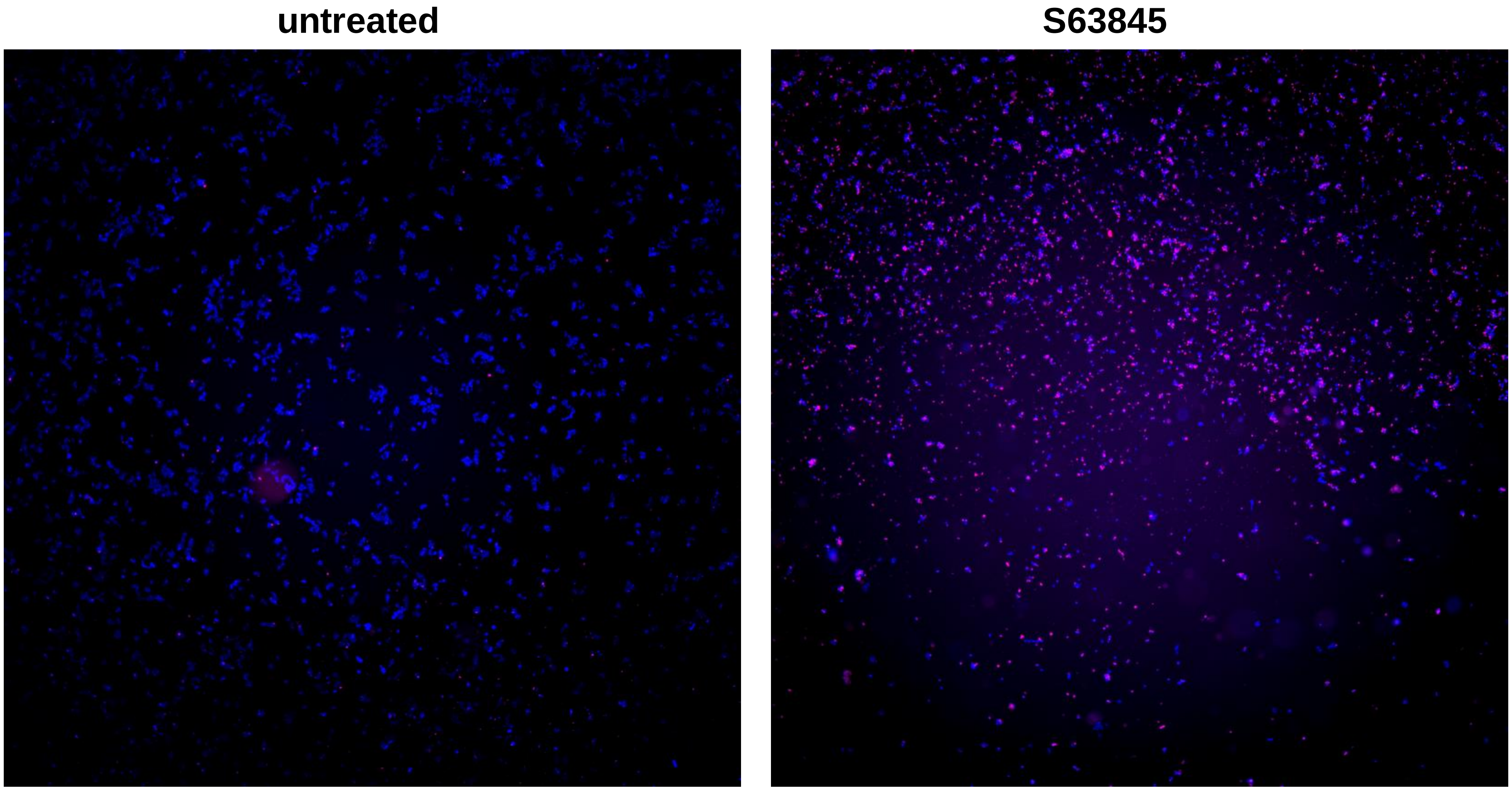

Supplementary Figure 1: Example of ImageXpress PI/HOECHST staining
Kelly cells were either left untreated or exposed to 1 μM S63845 for 48 h before staining with 1 μg/ml propidium iodide (red) and 10 μg/ml Hoechst33342 (blue) for 10 min. Cells were imaged using ImageXpress Micro XLS at 4x magnification using Cell Scoring application.

Supplementary Figure 2

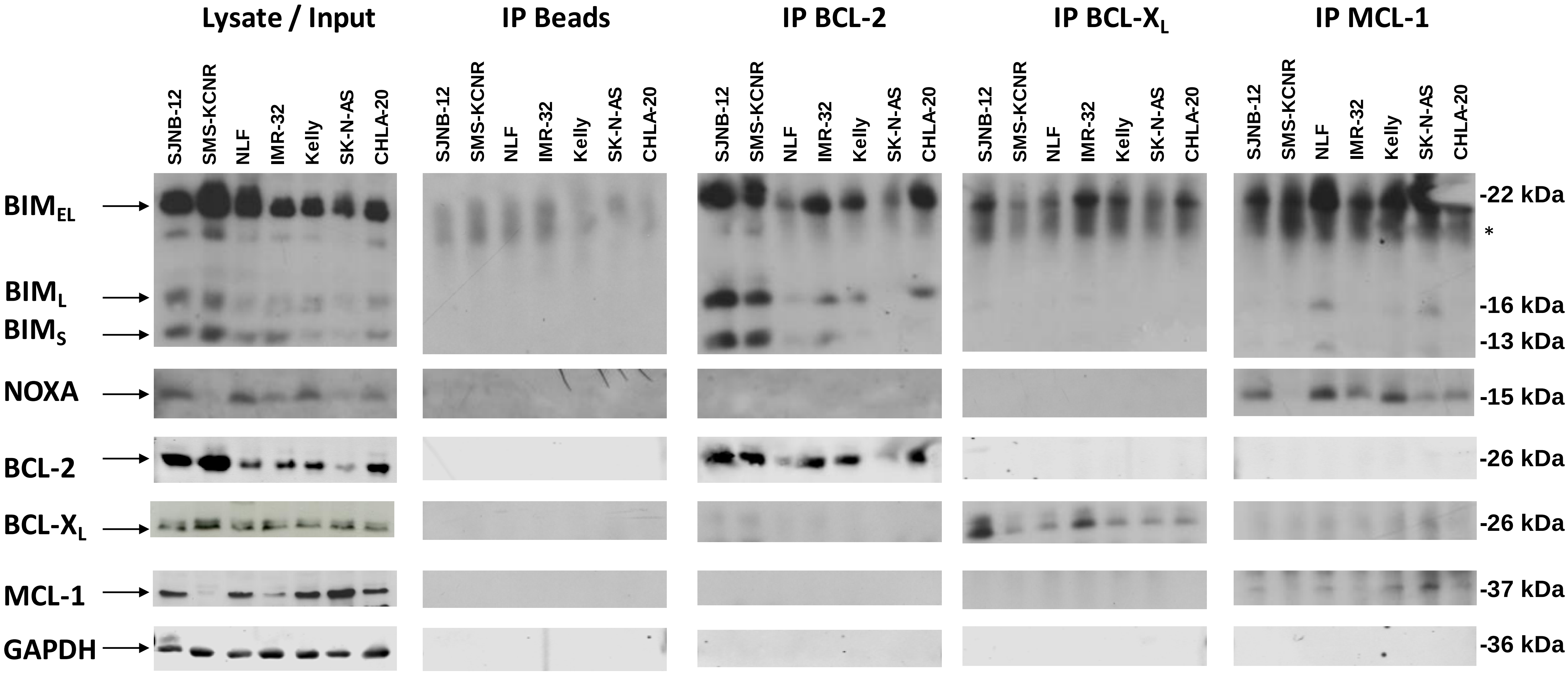

Supplementary Figure 2: Interaction of pro- and antiapoptotic BCL-2 proteins reveals high priming
Interaction of pro- and antiapoptotic BCL-2 proteins was investigated by IP of the main antiapoptotic BCL-2 proteins BCL-2, BCL-X_L and MCL-1. Western blotting was performed to detect bound proapoptotic BCL-2 proteins. BIM is expressed in the three isoforms BIM_{EL}, BIM_L and BIM_S.* indicates IgG band.

Supplementary Figure 3

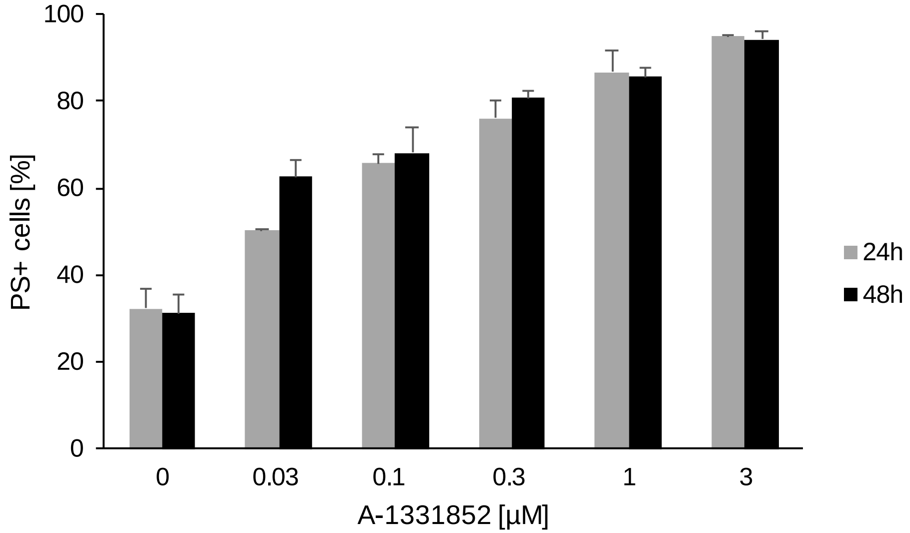

Supplementary Figure 3: Treatment with A1331852 induces phosphatidylserine (PS)-exposure
IMR-32 cells were treated with different concentrations of A1331852 for 24 or 48 h before staining with AnnexinV-FITC and analysis of PS-exposure by flow cytometry. Data shown are mean * S.D. (n=3).

Supplementary Figure 4

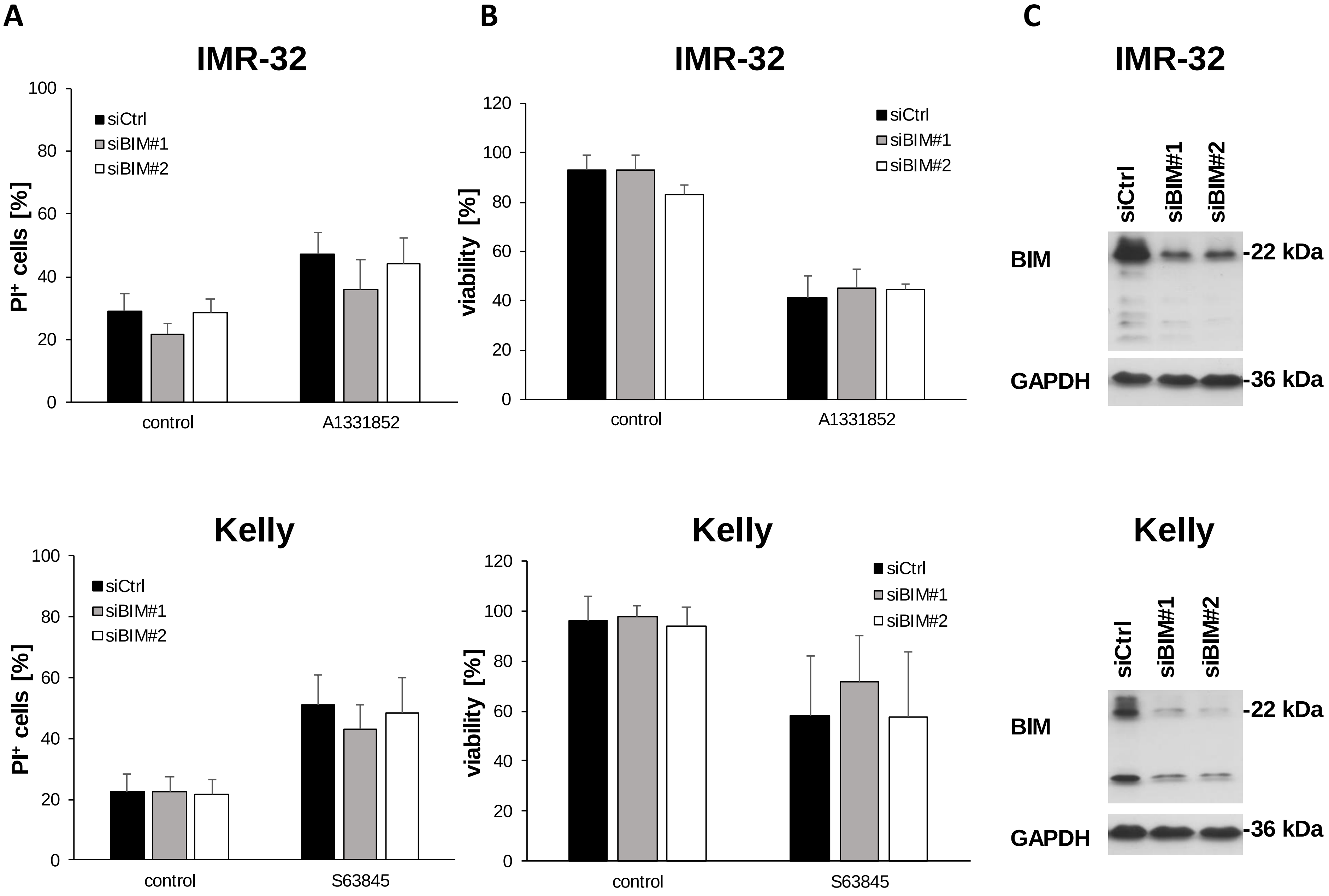

Supplementary Figure 4: Silencing of BIM does not prevent BH3-mimetic-induced apoptosis
Silencing of BIM with two distinct siRNAs was followed by treatment with A1331852 (IMR-32, upper panels) or S63845 (Kelly, lower panels) for 24 h and analysis of cell death by PI uptake and microscopy (A) or viability (B). Data shown are mean + S.D. (n= 4-6). Knockdown efficiency was controlled by Western blotting (C). GAPDH is shown as loading control.
